# Errors, Typographical, Et Cetera

**Published:** 1848-04

**Authors:** 


*Errors, Typographical, et cetera.*—One of the most fruitful sources of anxiety and vexation incident to assuming the responsibility of publications of any kind, is the frequent occurrence of errors which the reader will be likely to impute to carelessness, if not to ignorance. The journalist, especially, is exposed to annoyances of this description, having generally less leisure and opportunity to provide against them, and hence, the most competent and practiced in this line of literary labor, is constrained to solicit the indulgent oversight of his readers. How much more dependent upon the leniency of criticism is one who can neither presume upon any particular qualifications, nor upon the advantages of much experience! But, more than all, the medical journalist must rely upon favorable oversight in this respect, when his editorial services are performed amidst the irregular and varied requisitions of his daily professional avocations. The latter of necessity claim precedence over superadded pursuits, and of their requiring character, and the utter impossibility of reducing them to any systematic arrangement so as to leave stated periods for literary, or other labors, every medical man, in active practice, must be fully aware. In so far as we are concerned, we have no reason to feel that our readers are not disposed to make ample ample allowance for all venial errors. We allude to this subject, chiefly, that we may have an opportunity to express the hope that our readers will not think that *all* the inaccuracies which may be detected in every number of our journal, are evidences of our own inability to discover them, when too late for corrrection. We are as far from asking any peculiar favors, as we are from assuming to be beyond the necessity of charitable consideration, but we would have it considered that the medical practitioner who ventures to incur the responsibility of editorial duties, must, necessarily, often commit his manuscript to the hands of the compositor without having had time for elaborateness of composition, or careful revision, and that the correction of proof is generally required to be done at short notice, and must be attended to frequently in intervals snatched from other pressing engagements, with which the attention is pre-occupied. We would farther intimate that the printer is responsible for a certain proportion of errors which it is his province to avoid, or rectify, and, also, for some which have been duly marked, but escape correction. Were it not for circumstances which we have mentioned, we assure our readers that we should not be guilty of the tautology involved in the phrase "chemico vital chemistry," nor should we allow *anœmia* to be printed *anœmia*, *alkalies*, *alkalis*, and other perversions of orthography, &c., which are readily enough discoverable without microscopical analysis. .

That all our errors are oversights we do not mean to imply, and, to be perfectly candid on the subject, we should confess, in bespeaking the indulgence of our patrons for venial faults, we are not without hopes that good nature may lead them to place the most charitable construction on errors of which we are unfortunately unconscious, and for which no valid apology can be rendered.

